# Numbness on the Medical Take: An Atypical Presentation of Guillain‐Barre Syndrome With Unilateral Paraesthesia and Rapid Progression to Bulbar Palsy: A Case Report

**DOI:** 10.1002/ccr3.9586

**Published:** 2024-12-29

**Authors:** J. Stanley, Q. Zhou

**Affiliations:** ^1^ Acute Medical Department University Hospital Bristol and Weston Bristol UK; ^2^ Bristol Medical School University of Bristol Bristol UK

**Keywords:** atypical neurology, bulbar palsy, Guillain‐Barré syndrome, numbness, paraesthesia

## Abstract

Guillain‐Barré syndrome (GBS) is characterized classically by progressive and symmetrical motor weakness and areflexia. We describe a case of GBS with initially preserved reflexes and power, leading to delayed diagnosis, who latterly required urgent ventilator support and plasmapharesis to highlight the importance of considering atypical presentations of this common condition.

**Summary**

- Patients with GBS can present with unilateral sensory abnormalities, with or without motor symptoms, which may be suggestive of stroke. It is important to revisit the diagnosis once symptoms are evolving and be vigilant for atypical presentations of GBS.
- We report this case to highlight the variety of presentations of GBS, including that some cases may not have hyporeflexia or motor symptoms at presentation and the importance of early recognition to prevent critical respiratory failure and death.
- A pragmatic approach is recommended to diagnose and manage patients with atypical GBS. When there is high clinical suspicion with pending airway compromise, early ventilator support with plasmapheresis can save lives and prevent functional disabilities.

## 1 Introduction

Guillain‐Barre syndrome (GBS) follows a typical pattern of preceding infection (found in up to 70% of cases—usually either respiratory or gastrointestinal [1]), immune stimulation, and development of clinical disease which usually peaks in severity within 4 weeks [2]. Diagnostic tests are unavailable for many subtypes and in those where they are available (anti‐GQ1b (anti‐GQ1b ganglioside antibody) in our case) the results are typically not available in a timely manner. Therefore, it is important that clinicians have a high level of suspicion based on clinical findings and history, particularly for atypical presentations. This is particularly relevant given the clinical severity of GBS, with almost a quarter of patients requiring intubation and ventilation [3] and 3.9% of patients dying within 12 months [4].

GBS typically presents with motor symptoms, most commonly an ascending, symmetrical weakness and reduced or absent reflexes [2]. GBS exists on a continuum with Miller Fisher syndromes (MFS), which are typically associated with ataxia and ophthalmoplegia, in addition to the areflexia which is typical of both [5]. These conditions share the underlying pathophysiology of autoimmune phenomena targeted against peripheral nerves [2] with a typical monophasic disease course characterized by duration of onset to nadir of illness no greater than 28 days [5]. Given the absence of confirmatory diagnostic tests. GBS remains a diagnosis that rests heavily on clinical findings, in addition to the exclusion of other differential diagnoses through neuroimaging and the typical finding of “cytoalbuminologic dissociation” on lumbar puncture, (an elevated protein with a normal white cell count), which is present in 88% of cases by the end of the second week of illness [3].

In this case report, we will focus predominantly on GBS rather than MFS. Within the group of GBS three common variants to the typical presenting features exist [5]. First, the pharyngeal cervical‐brachial (PCB) weakness variant, first documented by Ropper in 1986 [6], which is characterized by weakness in the neck, shoulders, and oropharynx, without impairment of sensation. Second, bifacial weakness with paraesthesias, characterized by a bilateral facial nerve palsy, which can lead to diagnostic confusion with conditions such as Lyme disease, HIV, sarcoid, and syphilis, among others [7]. This variant has been documented to involve taste alteration [8]. Within this variant, sensory disturbance including paraesthesia is also reported [7], although in addition to weakness, rather than in isolation. Finally, the third common variant is the paraparetic variant, whereby individuals suffer back pain and bilateral leg weakness [9].

In GBS as a whole, up to 50% of patients experience some degree of back pain [10], which may predate the development of weakness, often making alternative diagnoses, including musculoskeletal injuries or nerve impingement appear more likely [11]. Additionally, autonomic instability can also be seen in over 50% of cases [10] and this can also cause diagnostic confusion, making common causes of shock such as dehydration or sepsis enter the diagnostic field.

We report this case to highlight the variety of presentations of GBS, including that some cases may not have hyporeflexia or motor symptoms at presentation and the importance of early recognition to prevent critical respiratory failure and death. A pragmatic approach is recommended to diagnose and manage patients with atypical GBS. When there is high clinical suspicion with pending airway compromise, early ventilator support with plasmapheresis can save lives and prevent functional disabilities.

## 2 Case Presentation

A 73‐year‐old female presented with a 12‐h history of left‐sided hand and foot paraesthesia and reduced mobility. She was admitted as a stroke thrombolysis call however, was deemed unsuitable for thrombolysis as she woke up with her symptoms. Prior to presentation, she had experienced 2 weeks of cough, fever, and muscle aches. These symptoms had subsided 2 to 3 days prior to presentation.

Observations were unremarkable. Her background included adenocarcinoma of the large bowel, surgically resected in 2015, cholecystectomy, cervical canal stenosis, and right foot drop. She was also under investigation for longstanding right arm weakness (latterly shown to be thoracic outlet obstruction and not of direct relevance to this case). Medications included citalopram, solifenacin, amlodipine, atorvastatin, and propranolol. No medications had been started or changed in the preceding month.

Initial examination revealed decreased sensation in the left hand to the level of the wrist and both forefeet in a nondermatomal distribution. There was no new motor impairment (longstanding right arm weakness noted). Muscle tone and tendon reflexes were normal throughout all four limbs. CT (computed tomography) brain with aortic arch to vertex angiogram did not reveal any evidence of bleed, stroke, or large vessel thrombus. Initial blood tests (Table 1) did not reveal a cause for her symptoms. A provisional diagnosis of atypical stroke was considered due to the acuity of onset of her symptoms and a plan was made to discharge with clopidogrel for outpatient TIA (Transient ischemic attack) clinic follow‐up, MRI (magnetic resonance imaging) brain and review of the diagnosis. However, she was unable to mobilize independently (despite normal power and reflexes in the lower limbs on examination) and was admitted for further investigation and support.

**TABLE 1:** Initial blood tests.

Test	Result (units)	Reference range (units)
CRP	4 (mg/L)	< 6 (mg/L)
WCC	8.87 (×10^9^/L)	4–11 (×10^9^/L)
Hb	161 (g/L)	120–150 (g/L)
Platelets	330 (×10^9^/L)	150–400 (×10^9^/L)
Urea	3.7 (mmol/L)	2.5–7.8 (mmol/L)
Creatinine	78 (μmol/L)	45–84 (μmol/L)
B12	235 (ng/L)	180–900 (ng/L)
Folate	9.8 (μg/L)	2.5–19.5 (μg/L)
TSH	1.16 (mIU/L)	0.27–4.2 (mIU/L)

In the early hours of day 3 of admission, she had an inpatient fall. On assessment, she was febrile and reported global weakness (both arms and legs). Examination demonstrated decreased sensation in all four limbs without the involvement of trunk and face. Subtle power reduction from 5/5 to 4/5 in all limbs was noted as compared to examination at presentation. An urgent MRI brain scan did not reveal a cause for her symptoms.

In the early hours of day 4, she deteriorated further, with difficulty managing her saliva and oxygen saturation of 70% (shown to be type 1 respiratory failure on arterial blood gas). She was rapidly intubated and ventilated in the local intensive care unit. Areflexia was noted on limb examination at this point. A clinical diagnosis of atypical Guillain‐Barre syndrome was made and plasmapheresis was initiated. Lumbar puncture demonstrated elevated protein (Table 2). Peripheral nerve conduction studies (NCS) demonstrated moderate sensory and motor polyneuropathy with a predominantly axonal underlying pathology (see Tables 3, 4, 5).

**TABLE 2:** Lumbar puncture results.

Test	Result
WBC	2/mm^3^
RBC	8/mm^3^
Glucose	4.94 mmol/L
Protein	0.95 g/L (reference < 0.54)
Culture	No growth 40 h
PCR enterovirus, HSV1, HSV2, VZV	Negative

**TABLE 3:** Sensory nerve conduction.

Nerve/Sites	Rec. site	Lat 1 ms	Amp μV
R Radial			
Forearm	Snuff box	2.3	9
R Sural			
Lower Leg	Ankle	NR	NR
R Median—Dig II, Dig III			
Dig III (Long)	Wrist	NR	NR

**TABLE 4:** Motor nerve conduction.

Nerve/Sites	Muscle	Lat 1 ms	Dur ms	Amp mV	Area %	Segments	Distance cm	Lat Diff ms	CV m/s
R Median—APB (abductor pollicis brevis)									
Wrist^a^	APB	6.1	7.5	3.9	100	APB—Wrist^a^			
Elbow	APB	11.4	7.5	3.5	104	Wrist^a^—Elbow	29	−5.3	55
R Ulnar—ADM (abductor digiti minimi)									
Wrist^a^	ADM	4.3	8.3	5.7	100	ADM – Wrist^a^			
B.Elbow	ADM	8.4	8.5	5.3	96.1	Wrist^a^—B.Elbow		−4.1	
R Peroneal—EDB (extensor digitorum brevis)									
Ankle^a^	EDB	7.7	10.6	2.3	100	EDB—Ankle^a^			
Fibula Head	EDB	15.8	10.2	2.1	106	Ankle^a^—Fibula Head	27	−8.1	33
Popliteal Fossa	EDB	18.7	9.1	1.7	65.2	Fibula Head—Politeal Fossa	9	−2.9	32
R Tibial—AH (Abductor hallucis)									
Ankle^a^	AH	8.6	10.6	1.8	100%	AH‐Ankle^a^			
Popliteal Fossa	AH	20.0	8.9	1.5	77.9	Ankle^a^—Popliteal Fossa	42	−11.4	37
R Peroneal—Tibialis anterior									
Fib Head	Tib Ant	4.8	13.7	3.1	100	Tib Ant—Fib Head			
Pop Fossa	Tib Ant	7.5	12.2	4.0	90.7	Fib Head—Pop Fossa	9	−2.7	34

^a^ Cold limb.

**TABLE 5:** *F* wave studies.

Nerve	*M* min ms	*F* min ms	Min *F*–*M* ms	Max *F*–*M* ms	Mean *F*–*M* ms
R Median—APB	6.6	32.3	25.7	28.6	27.2
R Ulnar—ADM	^a^				
R Peroneal—EDB	^a^				
R Tibial—AH	^a^				

^a^ Artifacts.

## 3 Diagnosis

The neurologists reviewed the case at their multidisciplinary meeting and felt that the absence of eye involvement meant Miller Fischer Syndrome (MFS) was unlikely and made a final diagnosis of “axonal variant GBS with bulbar palsy,” embracing the spectrum of both GBS and MFS (with the anti‐GQ‐1b and bulbar palsy reflecting some features of MFS and the sensory impairment, albuminocytological dissociation and latter development of areflexia reflective of GBS). The diagnostic criteria for GBS utilize the Brighton criteria, providing a level of diagnostic certainty based on clinical findings [3]. The criteria are summarized in Table 6, with certainty ranging from 1 (high) to 4 (low) based on the presence (+) or absence (−) of certain features. In this case, given the latter development of bilateral flaccid weakness and areflexia, the diagnostic certainty according to these criteria was level 1, the highest level of certainty. This highlights the fact that the presence of typical features, even if they are in addition to atypical features, is enough to make a diagnosis of GBS.

**TABLE 6:** Brighton classification criteria for GBS.

Diagnostic criteria	Diagnostic certainty			
	1	2	3	4
Bilateral, flaccid limb weakness	+	+	+	+/−
Decreased/absent deep tendon reflexes in weak limbs	+	+	+	+/−
Monophasic course. Onset‐nadir 12 h to 28 days	+	+	+	+/−
CSF cell count < 50/mL	+	+	−	+/−
CSF protein higher than normal value	+	+/−	−	+/−
NCS findings consistent with GBS subtype	+	+/−	−	+/−
Absence of alternative diagnosis	+	+	+	+

The axonal variant of GBS has been documented since 1978 and is characterized by axonal pathology on nerve conduction. Frequently the subtypes of axonal GBS are characterized by antibody detection [12], of which GQ1b, as found here, is one. However, typically the GQ1b‐related disorders are MFS, Bickersttaff brainstem encephalitis (BBE), or PCB. This case was not felt to be MFS due to the absence of ophthalmoplegia or ataxia. It was not felt to be BBE due to the absence of impaired consciousness or ophthalmoplegia typical of BBE [13]. It was not felt to be PCB due to the presence of lower limb weakness in addition to bulbar weakness.

Therefore, the diagnosis of “axonal variant GBS with bulbar palsy” was made, highlighting that this case straddles both the spectrum of typical GBS (limb weakness and reduced reflexes), axonal variant GBS with features of the PCB subtype (oropharyngeal weakness and axonal NCS) alongside highly atypical initial presenting features (asymmetrical paraesthesia and initial absence of weakness).

## 4 Follow‐Up

She underwent 7 cycles of plasma exchange and was extubated on day 9. Paraneoplastic and ganglioside antibodies were sent, of which anti‐GQ1b was positive. She was stepped down to the ward on day 11 and, following rehabilitation in hospital, was discharged to a rehab center, due to an ongoing need for physical assistance with transferring from seat to stand to bed. On day 31 after admission, she was discharged from hospital and able to function independently at home. On follow‐up review 8 months later, she had normal power in the left side of her body, with 4/5 power in her right arm and legs (right arm weakness longstanding, leg weakness felt likely residual to GBS). She has no further neurology follow‐up planned given that we do not suspect a recurrence of her condition.

## 5 Discussion

This case represents an atypical constellation of symptoms and diagnostic results under the umbrella of GBS and is presented to raise awareness of the spectrum of presentations with which GBS can manifest. The absence of weakness at initial presentation is very unusual, with one study estimating its occurrence in only 1% of cases [3]. The same study estimated that only 10% of patients display normal, as was the case here, or increased reflexes at presentation [3]. It should be noted that in the same study, all patients subsequently developed hyporeflexia of their legs [3], highlighting the importance of repeated clinical examination. In this case, repeated clinical examination at the time of deterioration and admission to the intensive care unit demonstrated progression to weakness and areflexia, making the diagnosis far clearer.

The described presentation of asymmetrical sensory disturbance in this case is also atypical. Pure sensory GBS (sensory deficits without motor involvement throughout the disease course) is controversial with only a small number of reported cases [14], which would not satisfy current diagnostic criteria for GBS [2]. Furthermore, when sensory symptoms occur without motor symptoms in GBS, as described in case reports, the sensory symptoms are typically symmetrical []. Indeed, the diagnostic criteria for GBS suggest that severe sensory signs with little or no weakness at onset should raise doubt about the diagnosis [16].

In typical GBS, only 1% of patients have normal NCS with 48% demyelinating and 6% axonal pathology (as in our case), 41% demonstrating abnormal NCS that did not fulfill criteria for either axonal or demyelinating, and 4% demonstrating unexcitable NCS [3]. As discussed in the diagnosis section, the finding of both axonal pathology and GQ1b antibodies can be indicative of MFS, BBE, and PCB [12]. As highlighted this case shares the most features with PCB but overlaps with typical GBS. A case series by Nagashima et al. of patients with “pure PCB” (weakness of the oropharyngeal, brachial, and cervical muscles with hyporeflexia in the absence of lower limb weakness or ophthalmoplegia) highlighted sensory disturbance in 60% of patients [17]. Given that our case also involved sensory disturbance it is possible that this is an atypical case of PCB, however we do not satisfy the diagnostic criteria specified in this paper for pure PCB of “no weakness in the legs.” Of the 100 patients included by Nagashima et al. however, 48 had PCB‐GBS overlap, representing that these conditions exist on a spectrum and that overlap diagnoses, such as ours, are frequent. In addition, only 39% of patients with PCB in this series had a positive result for anti‐GQ1b [17], as was found in our case, demonstrating that the presence or absence of antibodies alone does not help to rule in or out the diagnosis, even when considering atypical variants.

This case is presented to highlight that GBS should be considered as a differential diagnosis when a patient's clinical symptoms evolve and are not in keeping with the initial impression of common neurological conditions, such as stroke in this case. It is important to note that there is a wide spectrum of neurological symptoms manifested in patients with GBS. Therefore, careful documentation of examination results with regular assessment is essential to ensure that the initial diagnoses are re‐visited and appropriate treatments are instigated in a timely manner to improve clinical outcomes and patients' safety. As a final note, we are grateful to the patient involved for her permission to share her case. We have made all efforts to preserve confidentiality but accept that there remains a risk of deductive disclosure given the rareness of the clinical syndrome.

## Author Contributions

**J. Stanley:** project administration, writing – original draft. **Q. Zhou:** supervision, writing – review and editing.

## Consent

Written consent has been provided by the patient. They gave consent for this case to be written up and submitted to a journal for publication. The consent form has been shared with the journal.

## Conflicts of Interest

The authors declare no conflicts of interest.

## Data Availability

The authors have nothing to report.
